# Dynamic frailty risk assessment among older adults with multiple myeloma: A population-based cohort study

**DOI:** 10.1038/s41408-023-00843-5

**Published:** 2023-05-10

**Authors:** Hira Mian, Tanya M. Wildes, Ravi Vij, Matthew J. Pianko, Ajay Major, Mark A. Fiala

**Affiliations:** 1 Department of Oncology, McMaster University, Hamilton, Canada; 2 Division of Hematology/Oncology, University of Nebraska Medical Center, Omaha, NE USA; 3 Department of Medicine, Division of Medical Oncology, Washington University School of Medicine, St. Louis, MO USA; 4 Division of Hematology/Oncology, Department of Internal Medicine, University of Michigan Medical School, Ann Arbor, MI USA; 5 Division of Hematology, Department of Medicine, University of Colorado School of Medicine, Denver, CO USA

**Keywords:** Myeloma, Health services

## Abstract

Multiple myeloma (MM) is a cancer of older adults and those who are more frail are at high risk of poor outcomes. Current tools for identifying and categorizing frail patients are often static and measured only at the time of diagnosis. The concept of dynamic frailty (i.e. frailty changing over time) is largely unexplored in MM. In our study, adults with newly-diagnosed MM who received novel drugs between the years 2007–2014 were identified in the Surveillance, Epidemiology, and End Results (SEER)-Medicare linked databases. Using a previously published cumulative deficit approach, a frailty index score was calculated at diagnosis and each landmark interval (1-yr, 2-yr, 3-yr post diagnosis). The association of frailty with overall survival (OS) both at baseline and at each landmark interval as well as factors associated with worsening frailty status over time were evaluated. Overall, 4617 patients were included. At baseline, 39% of the patients were categorized as moderately frail or severely frail. Among those who had 3 years of follow-up, frailty categorization changed post diagnosis in 93% of the cohort (78% improved and 72% deteriorated at least at one time point during the follow up period). In a landmark analysis, the predictive ability of frailty at the time of diagnosis decreased over time for OS (Harrell’s C Statistic 0.65 at diagnosis, 0.63 at 1-yr, 0.62 at 2-yr, and 0.60 at 3-yr) and was inferior compared to current frailty status at each landmark interval. Our study is one of the first to demonstrate the dynamic nature of frailty among older adults with MM. Frailty may improve or deteriorate over time. Current frailty status is a better predictor of outcomes than frailty status at time of diagnosis, indicating the need for re-measurement in this high-risk patient population.

## Introduction

Multiple myeloma (MM) is a non-curable cancer of older adults with significant morbidity and mortality. Frailty is increasingly recognized as a predictor for long- and short-term outcomes, with those who are more frail at risk for poorer survival and higher rates of toxicity and treatment discontinuation [1]. Identifying and tailoring treatment for frail older adults with MM remains an unmet need. As older adults represent a heterogeneous group with wide variation in functional status, several MM-specific frailty tools have been developed [2]. These include the International Myeloma Working Group (IMWG) Frailty Index [3], the Simplified Frailty Index [4], and the Revised Myeloma Co-morbidity Index [5]. Patients identified as frail using these indices have poorer outcomes both in clinical trials as well as real-world population cohorts [6, 7].

While incorporation of these tools in routine clinical settings represents an important step in identifying frail patients, many of the tools being utilized for frailty assessment have been developed to only measure frailty at one time point, most commonly at the time of diagnosis. Additionally, these tools consist of variables that may be less responsive to change, such as age and previous baseline comorbidities, and therefore may not allow for reassessment of how frailty many change over a short interval in a patient with MM. This was recently highlighted in a systematic review in which, although frailty was increasingly incorporated in clinical trials in the last 2 years including in the relapsed setting, longitudinal frailty assessments (i.e. frailty assessments conducted at more than one time point during the clinical care trajectory) occurred in <10% of the included studies [8].

Understanding both improvements and deteriorations in frailty over a patient’s disease trajectory may have important considerations in dynamic treatment delivery based upon a patient’s changing fitness status. This may include escalation of MM therapy to maximize disease control among patients with improved fitness status or de-escalation of therapy among those with worsened fitness status to minimize toxicity.

A cumulative deficit approach characterizes frailty by evaluating an individual’s health status as a proportion of ageing-associated deficits an individual has incurred [9, 10]. When measured at a single time point (e.g. at time of diagnosis), it identifies frail MM patients with an inferior overall survival (OS) [11, 12]. It also has the advantage of not incorporating chronological age alone as one of the variables that automatically categorizes someone as being frail, which is a key limitation of the some of the existing frailty measures. Additionally, this approach may allow for a more dynamic measurement of frailty as ‘deficits’ may drop off when not actively coded or new deficits may accumulate with increasing comorbidities or deteriorating functional status. Electronic frailty indices using a cumulative deficit approach have emerged as an important tool for identifying frail older adults through the electronic medical record [13, 14]. An electronic frailty index based on electronic health records from the United States VA health care system, for example, demonstrated increased rates of adverse events, treatment discontinuation, and worse progression free survival and overall survival in frail versus fit (or less frail) MM patients [15].

Overall, there is a paucity of data both in clinical trials as well as the real-world data evaluating how frailty status may change over time in MM. To fill this clinical and knowledge gap, using an administrative database and the cumulative deficit approach to categorizing frailty, we aimed to (1) evaluate the dynamic trajectory of frailty categorization (2) examine the relationship between frailty and survival and (3) identify factors associated with deterioration of frailty in a cohort of older adults with MM during the first 3 years following diagnosis.

## Methods

### Data source

The data source for this study was the SEER-Medicare linked database, which provides cancer registry data from 18 geographic areas, covering ~28% of the United States population, and is linked to billing claims for Medicare beneficiaries [16]. This linked database contains information regarding patient demographics, tumor characteristics, and survival for those with a cancer diagnosis who reside in the coverage area. This data source broadly represents the health care experience of patients in the United States diagnosed with cancer and who are insured through traditional fee-for-service Medicare plans. This study was performed under a protocol approved by the Washington University School of Medicine Human Subject Committee.

### Participants

Using the SEER-Medicare linked database, we identified older adults (age > 65) with MM (International Classification of Disease for Oncology [third edition] code 9732) receiving novel drugs (immunomodulatory drugs and/or proteosome inhibitors) for newly-diagnosed MM between the years 2007–2014. To ensure completeness of billing codes required for the calculation of frailty, participants without Medicare fee-for-service Part A (inpatient) and B (outpatient), from 1 year prior to MM diagnosis to 3 years post-diagnosis or death, were excluded. Patients without Medicare Part D (prescription) for the 12 months post diagnosis were also excluded as MM treatment could not be ascertained.

### Variables

Frailty was defined according to a previously validated frailty index based on the cumulative deficit definition [17]. The frailty index (possible range 0-1) was calculated using 31 age-related health deficits, identified using diagnostic and procedural codes (Supplementary Table S1). These domains capture important intersectional elements of frailty based upon widely studied and established model of frailty [18] and include co-morbidity, function, cognition and mood, and other elements. Frailty was calculated at MM diagnosis and again at each landmark interval at 1-, 2-, and 3-years after diagnosis. Variables used to calculate the frailty index for a given year were drawn from the prior 12 months. Deficits were allowed to drop off in subsequent years if the variable was not coded over a 12-month period, reflecting recovery or resolution of the condition. All patients were coded as having cancer at each landmark regardless of codes being present. At each landmark, patients were categorized into five pre-defined groups: nonfrail (0–0.10), prefrail (0.11–0.20), mildly frail (0.21–0.30), moderately frail (0.31–0.40) and severely frail (>0.4) as previously published [19].

### Statistical analysis

Patient baseline demographic factors were summarized using measures of central tendency and proportions for the entire cohort as well as for each frailty subgroup. Multivariate ordinal logistic regression was conducted to assess the association of baseline demographic and clinical factors with frailty status at the time of diagnosis. Variables of interest included: age, sex, race, Medicaid enrollment, as a proxy for socioeconomic status, and first-line anti-myeloma treatment, defined as treatment within the first 6 months post-diagnosis.

A Sankey diagram was created to show the proportion of patients in each frail subgroup at each landmark interval (1-yr, 2-yr and 3-yr post diagnosis). The proportion of patients surviving through the 3-year landmark who had a change in frailty status, either worsening, defined as any change to a higher frailty category from one landmark to the next, or improving, defined as any change to a lower frailty category, were summarized descriptively. Additionally, multivariate logistic regression was conducted to assess for variables associated with a worsening frailty status, defined as any change to a higher frailty category at 1 year following diagnosis. Patients who were severely frail at baseline or who died prior to 1 year were excluded from this analysis, as either their frailty classification could not worsen or could not be assessed, respectively.

OS was calculated from the time of diagnosis for each frailty subgroup and each landmark interval of 1-, 2- and 3-year post diagnosis. Multivariate cox-regression models were conducted to assess the association of frailty with overall survival at the time of diagnosis and at each landmark interval and model performance was accessed by receiver operator curves and Harrell C Indices. *P*-values <0.05 were considered significant. All analyses were performed using SAS Enterprise Guide 5.1.

## Results

**Fig. 1:**
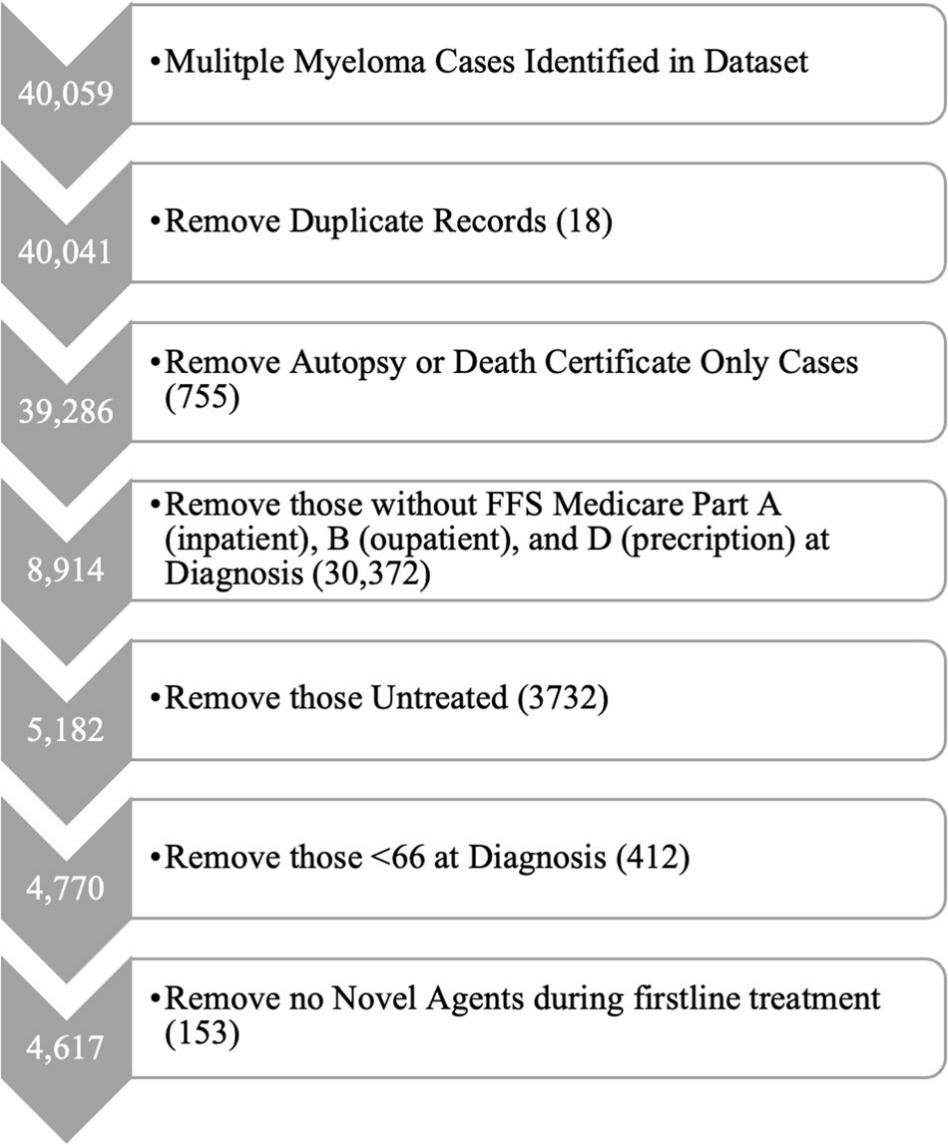
Cohort Selection for Study Inclusion.

A total of 4617 patient with MM receiving novel agents were identified based upon our inclusion criteria (Fig. 1). In the overall cohort, the median age was 75 with 81% of the cohort being of white race. With regards to frontline treatments, 68% and 59% of the cohort received a proteosome inhibitors and immunomodulatory agents, respectively.

At MM diagnosis, 214 (5%) were identified as non-frail, 1143 (25%) as pre-frail, 1479 (32%) as mildly frail, 1054 (23%) as moderately frail and 727 (16%) as severely frail. Baseline characteristics of the total cohort and for each category of fitness status are shown in Table 1. In multivariate analysis, a more recent MM diagnosis year, older age, female sex, Black race, and Medicaid enrollment were associated with an increased frailty status at the time of diagnosis (Supplementary Table S2). Immunomodulatory drug administration during first-line treatment was associated with lower baseline frailty.

**Table 1:** Baseline Characteristics of the included cohort.

	Total Cohort *N* = 4617	Nonfrail *N* = 214 (5%)	Prefrail *N* = 1143 (25%)	Mildly Frail *N* = 1479 (32%)	Moderately Frail *N* = 1054 (23%)	Severely Frail *N* = 727 (16%)	*P*-value
Age (median,IQR)	75 (70–80)	71 (68–75)	73 (69–78)	75 (70–81)	76 (71–81)	77 (72–83)	<0.01
MM dx Yr							0.02
2007	8%	8%	8%	8%	7%	6%	
2008	10%	11%	12%	10%	9%	6%	
2009	10%	11%	10%	10%	11%	9%	
2010	12%	15%	11%	12%	12%	11%	
2011	12%	8%	11%	12%	13%	13%	
2012	14%	12%	13%	16%	14%	15%	
2013	17%	16%	16%	15%	18%	20%	
2014	18%	20%	18%	17%	16%	19%	
Sex							
Male	52%	57%	57%	52%	50%	46%	<0.01
Race							
White	81%	86%	84%	81%	81%	77%	<0.01
Black	13%	6%	10%	13%	14%	16%	<0.01
Other Race	6%	7%	6%	6%	5%	7%	0.35
Medicaid Enrollment	23%	15%	15%	20%	29%	35%	<0.01
MM therapy							
PI	68%	65%	68%	67%	68%	70%	0.61
IMID	59%	62%	64%	61%	56%	50%	<0.01
Combination (PI + IMID)	27%	27%	32%	28%	24%	20%	<0.01

*IMID* Immunomodulatory Drug, *PI* proteosome inhibitor, *OS* overall survival, *dx* diagnosis.

**Fig. 2:**
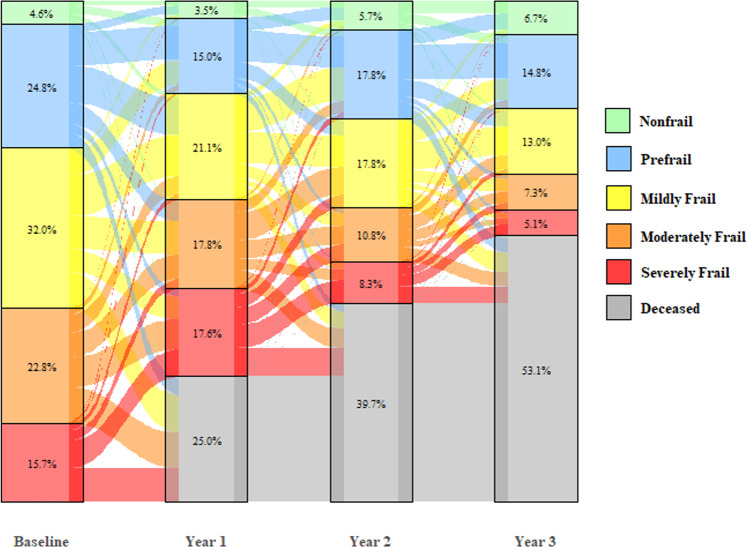
Trajectories of frailty in the first 3 year following diagnosis among older adults with MM.

Trajectories of frailty from baseline over the next 3 years is shown in Fig. 2. One year following diagnosis, 16% of the patients showed an improvement in the frailty status, 33% had a deterioration, 26% remained the same, and 25% had died. Subsequently, among the patients alive at the beginning of year 1 following diagnosis (*N* = 3462), 35% of the patients showed in an improvement in the frailty status, 17% had a deterioration, 29% remained the same, and 20% had died at the year 2 assessment. Among the patients alive at the beginning of year 3 (*N* = 2786), 25% of the patients showed an improvement in the frailty status, 20% had a deterioration, 32% remained the same, and 22% had died at the year 3 assessment. In total, among patient that were alive during the entire duration of the follow up 3 years (*N* = 2165), frailty categorization changed in 93% of the cohort post diagnosis;78% improved and 72% deteriorated at least at one time point during the follow up period.

**Fig. 3:**
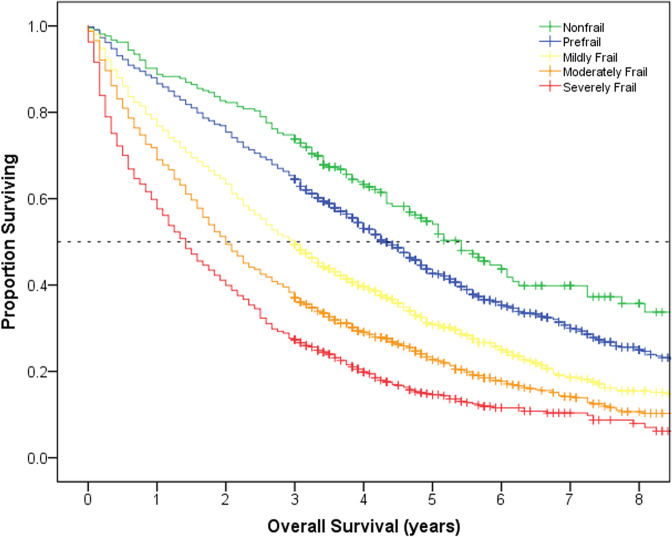
Overall Survival from the time of diagnosis according to baseline frailty status at diagnosis.

The median OS from the time of diagnosis for the total cohort was 34 months (95% CI 32-36). There was a clear gradient effect of frailty on OS (Fig. 3): the median OS for nonfrail patients was 65 months [95% CI 56-73] compared to 17 months [95% CI 15–19] for severely frail. At baseline, compared to nonfrail patients, the mildly frail had a 60% increase in hazard for death, the moderately frail a 104% increase, and the severely frail a 165% increase after controlling for other covariates (all *p* < 0.01, Supplementary Table S3). The predictive ability of frailty at the time of diagnosis decreased over time for OS in landmark analyses (Harrell’s C Statistic 0.65 at diagnosis, 0.63 at 1-year, 0.62 at 2-year, and 0.60 at 3-year, Supplementary Fig. 1). Conversely, landmark analyses at years 1-, 2- and 3- post diagnosis demonstrated that the predictive ability of the re-measured current frailty status at each of those time intervals remained fairly consistent for OS (Harrell’s C Statistic 0.64–0.65) and, overall, better predicted OS than the frailty status at the time of diagnosis.

As the largest change in frailty status occurred 1 year post-diagnosis, we conducted a multivariate analysis to determine factors associated with worsening frailty status. On multivariate analysis, older age, Medicaid enrollment and combination treatment with immunomodulatory drugs and proteosome inhibitors were associated with increased odds of worsening frailty status at 1 year (Table 2). Conversely, female sex, other race and immunomodulatory therapy alone were associated with a decreased odds of worsening frailty status.

**Table 2:** Multivariate logistic regression variables for predicting a worsening frail status at 1 year following diagnosis.

Demographics	aOR^a^	95% CI	*P*-value
MM diagnosis year (per yr)	1.01	0.97–1.04	0.78
Age (per year)	1.01	1.00–1.03	0.04
Sex			
Female	0.82	0.71–0.96	0.01
Race			
White	REF	REF	REF
Black	1.15	0.91–1.45	0.26
Other	0.64	0.46–0.89	0.01
Medicaid Enrollment	1.24	1.02–1.51	0.03
Frailty status at diagnosis*			
Nonfrail	REF	REF	REF
Pre-frail	0.50	0.35–0.72	<0.01
Mildly-frail	0.25	0.17–0.35	<0.01
Moderately frail	0.19	0.13–0.27	<0.01
MM therapy at diagnosis			
PI	REF	REF	REF
IMID	0.75	0.63–0.90	<0.01
Combination (PI + IMID)	1.56	1.28–1.90	<0.01

^a^aOR, odds ratio adjusted for other listed variables.

*Patients with severely frail status at baseline or who died within 1 year of diagnosis were excluded as frailty status could not further worsen or were not assessable for worsening frailty.

*IMID* Immunomodulatory Drug, *PI* proteosome inhibitor, *OS* overall survival, *dx* diagnosis.

## Discussion

Our study is one of the first to demonstrate the dynamic nature of frailty among older adults with MM and that frailty can either improve or deteriorate over time. Further, we demonstrate that current frailty status is a better predictor of outcomes than frailty status at the time of diagnosis, indicating the need for re-measurement of frailty over time in older adults with MM.

Frailty is now well-recognized to be a high-risk feature in MM. This has led to an increasing number of clinical trials incorporating frailty assessment, especially within the last 2 years. The majority of these trials measure frailty at one time point for study entry criteria or subgroup analysis, but seldom is frailty used for treatment delivery, which is currently being examined in the UKMRA FiTNEss study [20]. While the incorporation of frailty at any time point represents an important step in the evaluation of a patient, there is a paucity of data examining the longitudinal evaluation of frailty. To our knowledge, there is only one previously published therapeutic clinical trial (VBDD-VERRUM) and there are three ongoing studies evaluating how frailty changes over time (UKMRA FiTNEss, HOVON 123, HOVON 143) [8]. As compared to clinical trials, there is even less data in the real-world evaluating frailty over time. In this setting, our study results are novel demonstrating that not only does frailty change in the majority of patients, but can both improve or deteriorate over time, a concept previously unexplored among real-world MM patients. In the general geriatric literature (ie noncancer cohorts), data on changes in frailty over time are also sparse. One study of Taiwanese older adults over up to 8 years of follow-up demonstrated that most (69%) had a relatively stable frailty index in trajectory modeling over time, while 21.5% had a moderate increase in frailty index and 9.3% had a rapid increase in frailty index [21]. In another study that examined changes in frailty index as a continuous variable rather than ordinal, the mean change in frailty index was only 0.02 ± 0.07 over 1 year [22]. Most of these individuals would not likely have been reclassified based on the FI groupings we utilized. Altogether, these data suggest that the changes in frailty seen in the myeloma population are more rapid than seen in community dwelling older adult populations.

Observational studies are beginning to incorporate longitudinal assessment of frailty, but these studies are limited by single center evaluations and small numbers [6]. Our study uses a large population-based administrative database to evaluate the dynamic nature of frailty. In our study, the frailty categorization of over 93% of the patients changed (either improved or deteriorated) during the 3-year follow-up, with the largest change happening within the first year. Holler et al. did measure frailty at two time points with a median follow up of 11 months (range 6-24) among MM patients using the revised myeloma co-morbidity index. Similar to our study, they also noted changes in frailty with 43% showing improvement at the subsequent time point; however, their study was limited by re-measuring frailty at only one subsequent time point and the timing was inconsistent [23]. To our knowledge, no previous analysis similar to this has been done in patients with multiple myeloma specifically focusing on frailty changes at multiple time points during the treatment trajectory. While such an analysis had not been done in MM, our results are consistent with other studies done among community-dwelling older adults, which also demonstrate changes in frailty status over time [21, 22, 24].

Our study also demonstrated that current frailty status is a better predictor of outcomes compared to baseline frailty status assessed at the time of diagnosis. Although our study only evaluated frailty longitudinally among newly-diagnosed patients, frailty re-assessment at the time of each MM relapse may be particularly important. Several post-hoc analysis of recently conducted clinical trials in MM have demonstrated the prognostic value of frailty assessments in both newly-diagnosed and relapsed/refractory settings, even when retrospectively derived using the simplified frailty scale (modified IMWG frailty scale) [8]. Although, compared to the fit patients, patients who were more frail had poorer outcomes in these relapsed trials, many of these analyses were done only at a single time point and therefore additional studies are required to further understand the longitudinal trajectory of frailty in both the newly-diagnosed as well as the relapsed/refractory setting.

With regards to factors that predict worsening of frailty status 1 year following diagnosis, there is a paucity of data in this field, especially in MM. While chronological age did emerge as one factors for a deterioration in frailty status 1 year following diagnosis, female sex emerged as a potential protective factor for frailty deterioration. Studies conducted in general population studies have suggested that females tend to experience higher frailty at all stages despite having a lower risk of mortality [25]. Therefore, it is possible that given the higher proportion of females in the more frail categories from the time of diagnosis in our cohort, there was less further deterioration noted in our frailty scale. However, data is emerging that women may experience lower rates of transitions in frailty than men [26]. Additionally, Medicaid enrollment also emerged as an additional factor for frailty deterioration. Medicaid enrollment is an often used proxy for socioeconomic status [27] and has also been associated with reduced access to care for among people with multiple myeloma [28, 29]. It is possible that worsening frailty status with increased enrollment in Medicaid may be on the basis of unmeasured socioeconomic differences among the populations.

With regards to therapy, single agent immunomodulatory therapy was noted to be protective against further deterioration in frailty. While we controlled for baseline frailty status in the model, this may still reflect residual confounding by indication, that more frail individuals, who have lower odds of worsening, received an immunomodulatory drug alone rather than a proteosome inhibitor or combination therapy. Relative to patients receiving a proteosome inhibitor alone, those receiving a combination of an immunomodulatory agent and a proteosome inhibitor had a higher odd of increased frailty. The exact reason for this cannot be elucidated from our study as it is possible that those being initiated on combination have more aggressive disease or experiences higher treatment-related toxicities leading to a deterioration in frailty status at 1 year. Another possibility is that the greater toxicity of the combination accelerated frailty. Unfortunately, we are unable to reliably ascertain either progression or toxicity in this administrative dataset.

Strengths of our study include a large population-based analysis evaluating the concept of dynamic frailty, which remains a largely unexplored area in MM risk stratification. Limitations of our study are as follows. Although our study population is largely representative of the Medicare eligible population, the majority of patients are Caucasian which may limit the generatability of results to other racial and ethnic groups. Given the lack of specific patient- and disease- factors, we cannot comment on the reasons for change in frailty status over time. It is unknown, for example, whether a deteriorating frailty status was related to aggressive disease, suboptimal treatment, treatment toxicity or additional patient comorbidities unrelated to their malignancy. The impact of dexamethasone on frailty, both its receipt and potential dosing, for example, is not available in our cohort. Additionally, our study utilized a specific frailty assessment derived from administrative claims data using a cumulative deficit approach, which allowed for retrospective evaluation of deficits including both a combination of co-morbidities, functional status and cognitive/mood deficits. Due to inherent limitations of administrative databases and the retrospective nature of this study, it is possible that certain deficits may not meet the threshold for being coded as a deficit or maybe incorrectly coded but may still have an impact on frailty. Furthermore, it is unknown whether similar changes in frailty can be detected using more MM-specific assessment tools, such as the IMWG frailty index, which consists of chronological age, prospective evaluation of co-morbidities, and functional status. Similarly, it is unknown how changes detected in frailty, either improvement or deterioration, as noted by our frailty assessment scale would translate into minimum clinically important differences in impacting treatment decision making and patient outcome. However, our study highlights the needs for the development of frailty assessment tools which are sensitive, specific and responsive to change over time. Lastly, our study only explored the prognostic value of frailty for the outcome of OS; additional outcomes important to older adults, such as quality of life and functional independence, were not available in our databases.

In conclusion, our study demonstrates that frailty status itself may be dynamic and change over time. Additionally, current frailty status is a better predictor of outcomes than baseline frailty status at time of diagnosis, necessitating the need for re-measurement. Future strategies aimed at further assessing and incorporating dynamic frailty monitoring over time are needed to provide more tailored treatment among older adults with MM.

## Supplementary information

BCJ checklist

Supplement

## Data Availability

The data that support the findings of this study are available from the SEER-Medicare program, but restrictions apply to the availability of these data, which were used under license for the current study, and so are not publicly available. Data are however available from the authors upon reasonable request and with permission of the SEER-Medicare program.
