# Supplementary material for: Dynamic frailty risk assessment among older adults with multiple myeloma: A population-based cohort study
Source: Blood Cancer J. 2023 May 10;13(1):76. doi: 10.1038/s41408-023-00843-5 (PMC10172354; doi:10.1038/s41408-023-00843-5)
Supplement: Supplementary file 2 — Supplement [file 41408_2023_843_MOESM2_ESM.docx]

**Supplement**

Supplementary Table S1: List of Variables used to calculate the cumulative deficit frailty index at each index time point

| Morbidity | | | |
| --- | --- | --- | --- |
| Anemia | Atrial Fibrillation | Cancer (except basal cell skin cancer) | Cerebrovascular disease: Stroke/TIA |
| Coronary Artery Disease: MI/CABG/PCI | Diabetes | Heart Failure (diastolic or systolic) | Hypertension |
| Kidney Disease | Liver Disease or Cirrhosis | Lung disease: COPD or Asthma | Thyroid Disease |
| Osteoporosis or osteoporosis related fracture | Incontinence |  |  |
| Functions | | | |
| Arthritis (rheumatoid arthritis or osteoarthritis) | Use of Durable Medical Equipment | Fall or Fall related diagnoses:  Hip fractures/  subdural hematoma/ subarachnoid hematoma | Fatigue |
| Gait Abnormality or difficulty walking | Parkinson’s Disease and Tremors | Peripheral vascular disease or Intermittent claudication | Muscular wasting and disuse atrophy/ Cachexia/Debility |
| Hearing Impairment/  Hearing Aid | Peripheral Neuropathy | Vision Comorbidity (macular degeneration, glaucoma, blindness) |  |
| Cognition and Mood | | | |
| Dementias (Alzheimer’s, Vascular, Lewy Body, Pick’s disease, Mild Cognitive Impairment, etc.) | Anxiety | Depression/Bipolar |  |
| Other | | | |
| Chronic Pain | Failure to thrive | Weight loss |  |

Supplementary Table S2: Multivariate ordinal logistic regression of variables of association between baseline variables and frail status at the time of diagnosis

| Demographics | aOR^ab^ | 95% CI | P-value |
| --- | --- | --- | --- |
| MM diagnosis year  (per year) | 1.05 | 1.02-1.07 | <0.01 |
| Age (per year) | 1.06 | 1.05-1.07 | <0.01 |
| Gender  Female | 1.19 | 1.07-1.32 | 0.01 |
| Race  White  Black  Other | REF  1.22  0.84 | REF  1.03-1.43  0.67-1.05 | REF  0.02  0.12 |
| Medicaid Enrollment | 2.14 | 1.88-2.44 | <0.01 |
| MM therapy at diagnosis  PI  IMID  Combination (PI+IMID) | REF  0.73  0.99 | REF  0.65-0.83  0.86-1.15 | REF  <0.01  0.93 |
| ^a^aOR, odds ratio adjusted for other listed variables | | | |

Supplementary Table S3: Multivariate cox regression model of variables associated with overall survival among adults with newly-diagnosed MM

| Demographics | aHR^a^ | 95% CI | P-value |
| --- | --- | --- | --- |
| MM diagnosis year (per year) | 0.96 | 0.95 | <0.01 |
| Age (per year) | 1.04 | 1.04 | <0.01 |
| Sex  Female | 0.81 | 0.75 | <0.01 |
| Race  Black  Other | 1.09  0.77 | 0.98  0.66 | 0.12  <0.01 |
| Medicaid Enrollment | 1.13 | 1.04 | <0.01 |
| Frailty status at diagnosis  Pre-frail  Mildly frail  Moderately frail  Severely frail | 1.20  1.60  2.04  2.65 | 0.99  1.32  1.67  2.16 | 0.07  <0.01  <0.01  <0.01 |
| MM therapy at diagnosis  PI  IMID  Combination (PI+IMID) | REF  0.92  0.89 | REF  0.85  0.81 | REF  0.03  0.02 |
| ^a^aOR, odds ratio adjusted for other listed variables | | | |

Supplementary Figure 1: ROC Curves for Survival Comparing Baseline and Contemporary Frailty Assessment


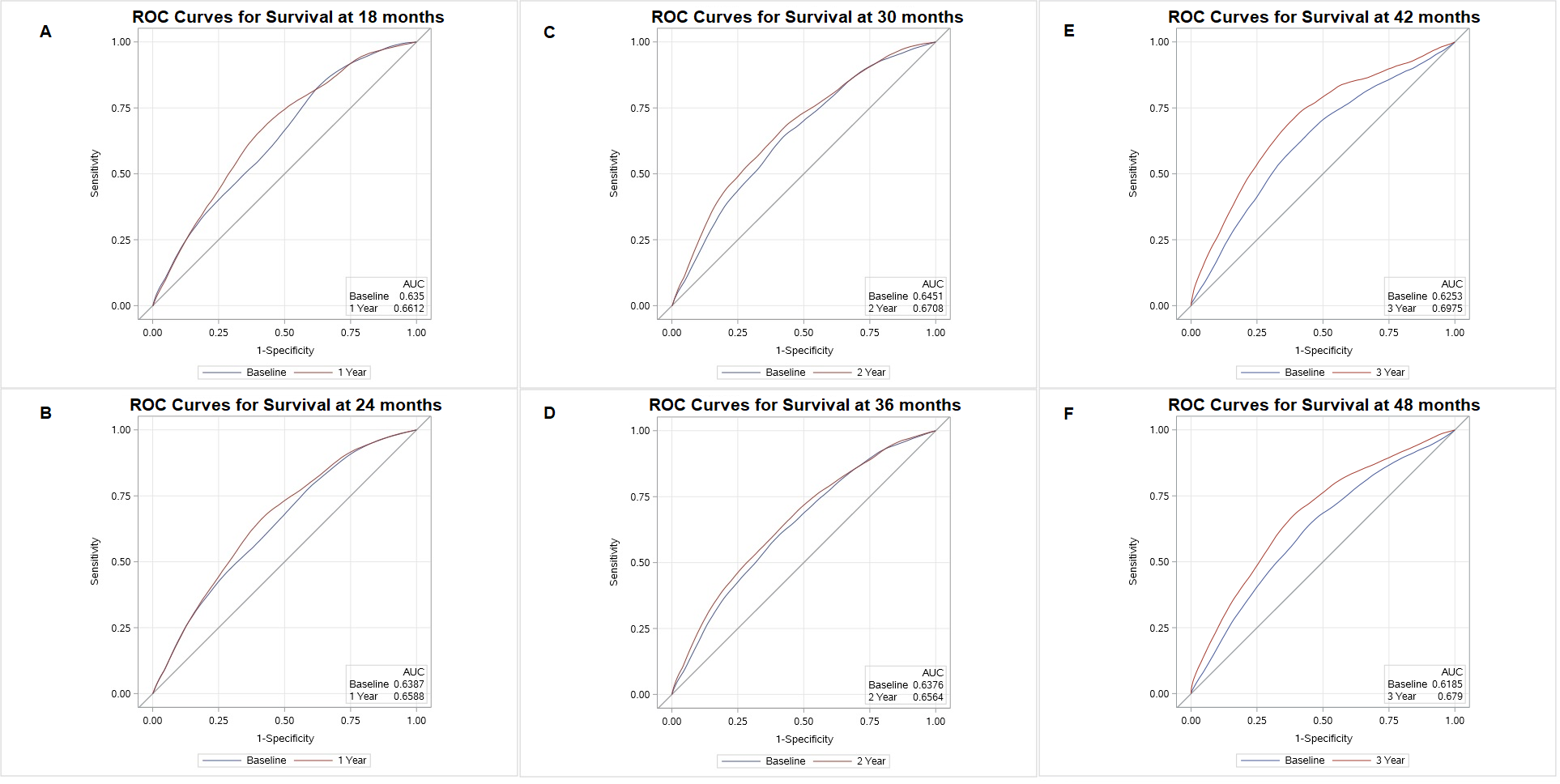


Figure Caption: ROC curves demonstrate the prediction of contemporary frailty assessment compared to baseline. At 18 months and 24 months the 1 year assessment performed better than baseline (A and B). At 30 months and 36 months the 2 year assessment performed better than baseline (C and D). At 42 months and 48 months the 3 year assessment performed better than baseline (C and D). For all analyses, patients who died prior to the follow-up assessment were excluded.
